# Flavonoids as Potential Anti-Inflammatory Molecules: A Review

**DOI:** 10.3390/molecules27092901

**Published:** 2022-05-02

**Authors:** Jameel M. Al-Khayri, Gandasi Ravikumar Sahana, Praveen Nagella, Biljo V. Joseph, Fatima M. Alessa, Muneera Q. Al-Mssallem

**Affiliations:** 1 Department of Plant Biotechnology, College of Agriculture and Food Sciences, King Faisal University, Al-Ahsa 31982, Saudi Arabia; 2 Department of Life Sciences, CHRIST (Deemed to be University), Hosur Road, Bangalore 560029, India; gr.sahana@res.christuniversity.in (G.R.S.); biljo.joseph@christuniversity.in (B.V.J.); 3 Department of Food Science and Nutrition, College of Agriculture and Food Sciences, King Faisal University, Al-Ahsa 31982, Saudi Arabia; falissa@kfu.edu.sa (F.M.A.); mmssallem@kfu.edu.sa (M.Q.A.-M.)

**Keywords:** anti-inflammation, flavonoids, natural products, phenolic compounds, phytochemicals

## Abstract

Hydroxylated polyphenols, also called flavonoids, are richly present in vegetables, fruits, cereals, nuts, herbs, seeds, stems, and flowers of numerous plants. They possess numerous medicinal properties such as antioxidant, anti-cancer, anti-microbial, neuroprotective, and anti-inflammation. Studies show that flavonoids activate antioxidant pathways that render an anti-inflammatory effect. They inhibit the secretions of enzymes such as lysozymes and β-glucuronidase and inhibit the secretion of arachidonic acid, which reduces inflammatory reactions. Flavonoids such as quercetin, genistein, apigenin, kaempferol, and epigallocatechin 3-gallate modulate the expression and activation of a cytokine such as interleukin-1beta (IL-1β), Tumor necrosis factor-alpha (TNF-α), interleukin-6 (IL-6), and interleukin-8 (IL-8); regulate the gene expression of many pro-inflammatory molecules such s nuclear factor kappa-light chain enhancer of activated B cells (NF-κB), activator protein-1 (AP-1), intercellular adhesion molecule-1 (ICAM), vascular cell adhesion molecule-1 (VCAM), and E-selectins; and also inhibits inducible nitric oxide (NO) synthase, cyclooxygenase-2, and lipoxygenase, which are pro-inflammatory enzymes. Understanding the anti-inflammatory action of flavonoids provides better treatment options, including coronavirus disease 2019 (COVID-19)-induced inflammation, inflammatory bowel disease, obstructive pulmonary disorder, arthritis, Alzheimer’s disease, cardiovascular disease, atherosclerosis, and cancer. This review highlights the sources, biochemical activities, and role of flavonoids in enhancing human health.

## 1. Introduction

**Figure 1:**
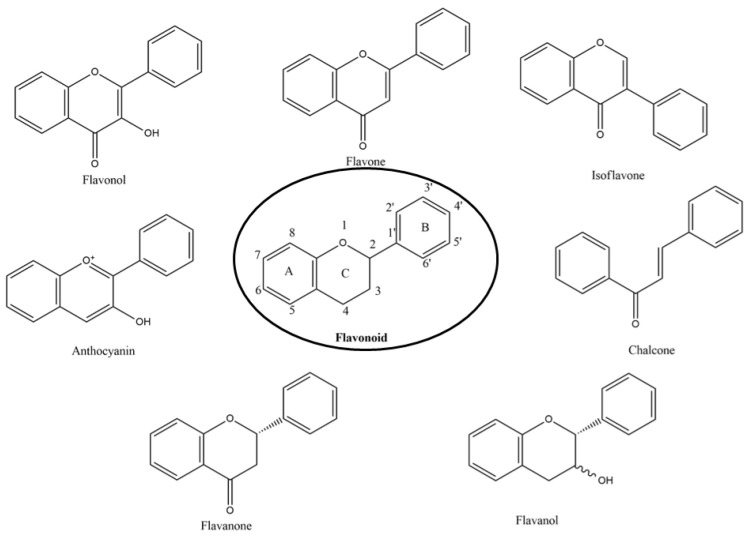
Structure of different groups of flavonoids.

Nutrition is one of the major aspects required to lead a healthy life. A balanced diet includes plant-based foods and contains non-starch polysaccharides, bioactive compounds, poly/monounsaturated fatty acids, and polyphenols. Polyphenols are a class of compounds that are produced by plants as secondary metabolites and play an important role in reproduction and pigmentation and also help plants in fighting pathogens. Although there are many polyphenols, up to 8000 are identified as flavonoids [1]. Flavonoids are hydroxylated polyphenols with two or more aromatic rings connected by heterocyclic pyran and at least one aromatic hydroxyl group attached (Figure 1) [2]. They exist in either glycosylated form or as aglycones [3]. Epidemiological studies show that flavonoid-rich foods prevent some diseases, including metabolic-related diseases and cancer. Further studies show that flavonoids have many properties, which include antioxidant, anti-inflammatory, analgesic [4], anti-proliferative, anti-cancer, anti-angiogenic, anti-microbial, anti-viral, and anti-malarial properties and also neuroprotective activity [5,6].

**Figure 2:**
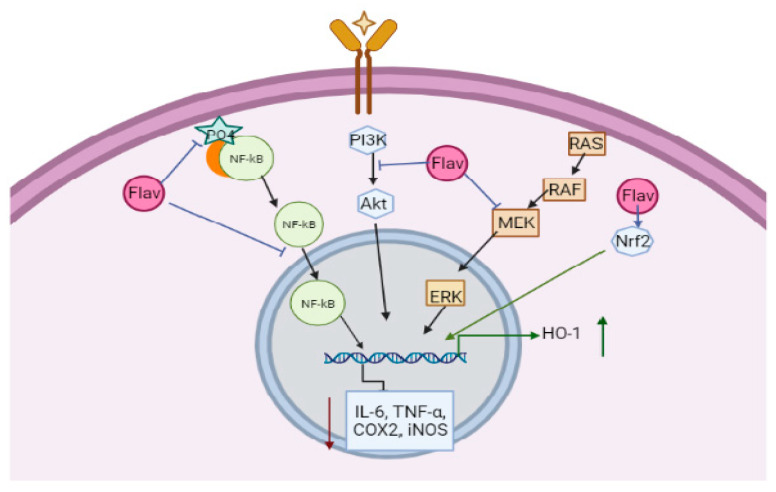
Major inflammatory pathways targeted by flavonoids.

Inflammation is a reaction to a foreign substance by the body. The immune cells of the body recognize different foreign bodies such as viruses, bacteria, parasites, antigenic substances, or chemicals using many cell receptors. Once recognized, many pro-inflammatory pathways are activated, which leads to the production of cytokines and the activation of immune cells, including macrophages and lymphocytes that eliminate foreign bodies. However, if the body fails to eliminate these foreign bodies in the early phase, inflammation increases, which is also called the chronic phase mediated by the overproduction of cytokines, chemokines, and inflammatory enzymes. The inflammation is regulated by many receptor-mediated pathways, which include Toll-like receptors, mitogen-activated protein kinase (MAPK) pathway, and the nuclear factor kappa-light chain enhancer of activated B cells (NF-κB), which is known to regulate more than 50 genes related to inflammation (Figure 2) [7]. The NF-κB pathway regulates the expression of the cyclooxygenase 2 (COX2) enzyme and many cytokines, which further activate endothelial cells. The further cascade of signalling is carried out to attract neutrophils, which releases prostaglandin E2 (PGE2) using COX1 or 2 enzyme, cytokines, reactive oxygen species (ROS), and histamines to induce inflammation and pain (Figure 2) [8,9]. The deregulation of these pathways leads to many inflammatory disorders such as vascular proliferation, tissue destruction, fibrosis, and many other secondary diseases such as arthritis, atherosclerosis, cardiovascular diseases, Alzheimer’s disease, asthma, and cancer [10]. Flavonoids with anti-inflammatory properties can interact with many molecules involved in inflammatory pathways and decrease the activity of cytokines, chemokines and also inflammatory enzymes (Figure 2). The present review focuses on sources, the mechanisms of anti-inflammatory activities of different flavonoids, and their impact on human health.

## 2. Sources of Flavonoids

Fruits and vegetables of many plants are rich in flavonoids. They are also present in leaves and flowers of a few plants. Apples, grapes, citrus fruits, berries, tea, onions, olive oil, and red wine are the most common sources of flavonoids [1]. Flavonoids are characterised by a 15 carbon skeleton consisting of two benzene rings labelled as A and B rings connected by a heterocyclic pyran named C ring (Figure 1) [1,4]. Flavonoids are classified based on their structural differences, such as flavanones, flavones, isoflavones, flavonols, and anthocyanin (Table 1) [1,11]. The classes are differentiated based on the substitutions and degree of oxidation in heterocyclic pyran [1,4].

### 2.1. Flavones

Flavones are a subclass which lack oxygenation at C3 [6] and possess a double bond between C2 and C3 while also possessing a ketone group in C4 [12], but it might have other modifications such as hydroxylation, methylation, glycosylation, or alkylation (Table 1) [6]. Apigenin, wogonin, luteolin, and tangeretin are flavones which are rare and are present in sweet red pepper, parsley, chamomile, celery, mint, and *Ginkgo biloba* [11].

### 2.2. Flavanols

Flavanols or flavan-3-ols are 3-hydroxy derivatives of flavonones (Table 1) [12]. They include the simplest monomer catechins, epicatechin to structurally complex gallocatechin, epigallocatechin, epicatechingallate, epigallocatechingallate, and procyanidin [6]. These are commonly present in black and green tea and fruits such as bananas, peaches, blueberries, apples, etc. (Table 2) [11].

### 2.3. Flavonols

Kaempferol, myricetin, fisetin, silymarin, rutin, isorhamnetin, and quercetin are flavonols, and they are present in saffron, lettuce, tomatoes, apples, grapes, berries, onions, kale, red wine, and tea (Table 2) [11]. These flavonols are characterised by the presence of both ketone group and hydroxyl group in C3 (Table 1). Flavonols are commonly found in conjugated form than as aglycones [6].

### 2.4. Flavanones

Naringenin, taxifolin, eriodictyol, and hesperetin are flavanones present in citrus fruits [1], and they are responsible for the bitter taste of the citrus peels. They are very similar to flavones but differ only by having saturated C-ring (Table 1) [12].

### 2.5. Isoflavones

Isoflavones, also called phytoestrogen as they share a similar structure with oestrogen, are also a class of flavonoids which have a B-ring attached to C3 position (Table 1). Daidzein and genistein are isoflavones present mainly in leguminous plants such as soy [6]; soy products such as tofu, roasted soya nuts, and miso are also rich in isoflavones [11].

### 2.6. Anthocyanins

Cyanidin, pelargonidin, malvidin, delphinidin, and peonidin are anthocyanins, which are responsible for imparting colour to fruits and flowers. The colour differs based on the conjugation at A or B rings of anthocyanins (Table 1) [12]. They are present mainly in outer cell layers of many fruits such as merlot grapes, red grapes, raspberries, strawberries, blueberries, cranberries, bilberries, and blackberries [11].

## 3. Biochemical Activities of Flavonoids

Flavonoids are exogenous antioxidants; they reduce reactive species by inhibiting nitric oxide (NO) synthase, xanthine oxide synthase, or regulating ion channels. They also modulate enzymes involved in oxidative mechanisms [5]. In addition, flavonoids reduce low-density lipoprotein (LDL) oxidation by peroxynitrite produced by the interaction of NO and superoxide ions induced by active macrophages [2]. Apigenin was also observed to decrease oxidative stress markers such as glutathione peroxidase, malondialdehyde, and superoxide dismutase and caspase-3 activation was also downregulated [11]. Luteolin-7-glucoside (LUTG) from *Ixeris chinensis* (Thunb.) Nakai showed substantial anti-oxidant properties [15]. It has been found that flavonoids and phenol compounds of dill and parsley plants reduced ROS [16]. Moreover, they enhance the glutathione-S-transferase (GST) enzyme, which is an antioxidant [17].

The hydroxyl group at specific positions in aromatic rings of flavonoids enhances antibacterial properties. Flavonoids are known to inhibit nucleic acid synthesis, energy metabolism, cell membrane function, biofilm synthesis, and pathogenesis, thereby attenuating the infection [18]. *Anisomeles malabarica* (L.) R. Br. ex. Sims extracts containing flavonoids showed anti-oxidant and antibacterial properties against *Bacillus subtilis, Staphylococcus aureus*, and *Proteus vulgaris* [19].

Some flavonoids such as wogonin and baicalin have shown both cytotoxic and cytostatic activities in tumour cells. Flavonoids derived from *Ouratea* species are good inhibitors of DNA topoisomerase, anti-viral, antibacterial, and antitumor agents [20]. Flavonoids also regulate many pathways such as MAPK, extracellular signal-regulated kinase (ERK), Phosphoinositide 3 kinase (PI3K)/Akt, and protein kinase-related pathways to reduce oxidative stress and inflammation, thereby acting as cardio-protective, neuroprotective, and anti-cancer agents [5]. Studies show that anthocyanins, flavanols, isoflavones, and morin hydrates can prevent heart disease. Flavonoid-rich Brazil nuts are shown to improve cardiovascular health and cancer [21]. Chrysin can reduce neuroinflammation, depression, and epilepsy [5].

**Figure 3:**
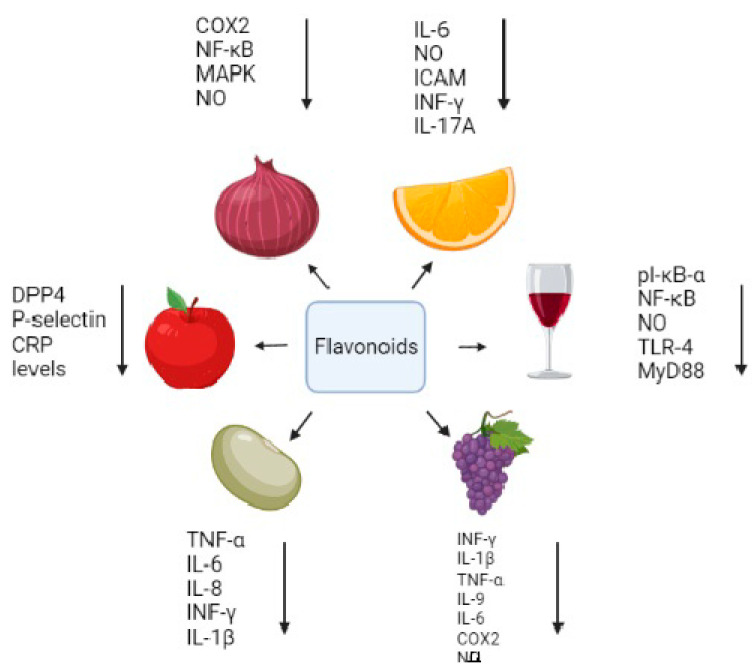
Different flavonoids and their function.

*Juniperus communis* L., a Romanian species, is observed to possess anti-microbial, anti-oxidant, and anti-inflammatory properties [22,23,24,25]. It was further studied that the extract of the plant had polyphenols, including terpenoids, phenolic acid, and flavonoids such as rutin (67.4 mg L^−1^), apigenin (13.2 mg L^−1^), and quercetin (11.2 mg L^−1^). The *Juniperus* extracts also have shown mitostimulatory properties, which were depicted by an increase in the mitotic index of root tips of *Allium cepa* L. [26]. In addition, flavonoids reduce inflammations by acting on many regulatory substances. These include the inhibition of NF-κB, activator protein-1 (AP-1), interleukin-1beta (IL-1β), Tumor necrosis factor-alpha (TNF-α), IL-6, IL-8, and COX2 (Figure 3). The anti-inflammatory activity of the flavonoid also renders protection against some diseases, which are elaborated in further sections.

## 4. Bioavailability of Flavonoids

The activity of flavonoids depends on the absorption and bioavailability of the compounds. Studies have shown that after absorption in the stomach and small intestine by either active transport or passive diffusion; flavonoids are metabolised in small intestinal cells and liver. The metabolism includes the biotransformation of flavonoids by the addition of sulphate, glucuronide, or methyl groups. Few flavonoids are also absorbed by the microflora in the colon, which breaks down flavonoids into phenolic acids and aldehydes. These are further transported into the blood stream after modifications in the liver [1].

The absorption and bioavailability of flavonoids differ by their structure, molecular weight, and their ability to undergo esterification glycosylation [27]. Flavonoids such as caffeic acid, quercetin, and p-coumaric acid are less abundant in circulation. It is shown in a study where 250 g of lettuce containing 31.7 mg caffeic acid, 12.7 mg quercetin, and 7.3 mg p-coumaric acid was ingested by healthy individuals and the concentration of different flavonoids at basal and after 3 h in serum was as follows: caffeic acid 34 µg/L at basal, 63 µg/L at 3 h; quercetin 46 µg/L basal, 66 µg/L at 3 h; and p-coumaric acid 46 µg/L basal, 85 µg/L at 3 h. This indicates the low availability of flavonoids in the serum [1]. Furthermore, quercetin was observed to be five times more bioavailable when administered in the form of quercetin glycoside than natural quercetin. Bioavailability also varied based on the food source containing quercetin. Onion powder extract provided greater bioavailability than apple peel powder. Citrus flavonones showed lower bioavailability, although absorption was faster [27].

The bioavailability of different flavonoids thereby has to be enhanced for better pharmaceutical activities. Different techniques are used such as microparticle [28], nanoparticle formulation [29], self-nanoemulsifying drug delivery system [30], liposomal vesicles [31], solid dispersions [32], inclusion complexes, and micelles [33] to deliver flavonoids or plant extracts to the site of action.

## 5. Role of Flavonoids as Anti-Inflammatory Agents

Flavonoids are known to have good anti-inflammatory properties. The structure of flavonoids plays an important role in anti-inflammatory activity. They have a planar ring structure with unsaturation at C_2_–C_3_, and the hydroxyl group positions are crucial to impart the property. The hydroxyl group at the 3’ and 4’ positions of the B-ring is very important, without which anti-inflammatory activity of the compound is lost [10]. In flavonoids, apigenin was shown to decrease the steady-state mRNA levels induced by TNF-α and thereby decreased the expression of intercellular adhesion molecule-1 (ICAM-1), E-selectin, and vascular cell adhesion molecule-1 (VCAM-1) on endothelial cells. It was also observed that cells pretreated with apigenin showed inhibitions of TNF-α-induced IL-1β, IL-6, and also prostaglandin E₂ (Table 3) (Figure 3) [1,34]. A study by Li et al. (2013) noted that many flavonoids decreased the expression of pro-inflammatory cytokines such as IL-6, IL-8, TNF-α, IL-1β, and monocyte chemoattractant protein-1 (MCP-1) in RAW macrophages, peripheral blood mononuclear cells, and Jurkat T cells (Figure 3) [35]. Catechins and quercetin could enhance IL-10 production, an anti-inflammatory compound, by the combined inhibition of IL-1β and TNF-α [1]. Quercetin, a flavonoid, blocks the activity of heat shock factor (HSF), which is required for the induction of heat shock protein (HSP) HSP70, hence reducing heat-induced damage [34]. Flavonols such as quercetin, morin, kaempferol, and myricetin exhibited lipoxygenase inhibition [1]. Many flavonoids are also potent inhibitors of arachidonic acid, phospholipase A₂, cyclooxygenase, and NOS (Figure 3). This reduces the production of prostaglandins, leukotrienes, and NO, which are key inflammatory substances [2]. Flavonoids also decreased the release of arachidonic acid metabolites and chemokines, thereby decreasing leukocyte infiltration and edema [34]. Moreover, flavonoids chelate iron and inhibit complement system activation, hence lowering inflammation [36]. They also chelate transition ions, hence reducing ROS generation [17].

**Figure 4:**
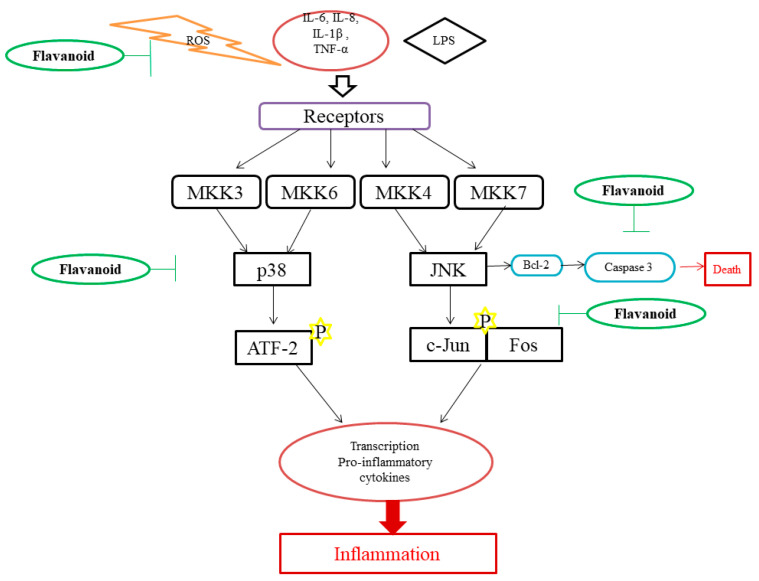
Anti-inflammatory action of flavonoids.

Genistein, a flavonoid, was shown to be effective in reducing pulmonary eosinophilia, ovalbumin-induced bronchoconstriction, and airway hyper-responsiveness in an asthma model of guinea pig. It also showed protective properties in rats against endotoxin-induced organ failure when injected intraperitoneally. It also reduced the inflammation and destruction of joints induced by collagen in arthritic mice. Rutin, hesperidin, and quercetin reduced acute and chronic inflammation in an experimental model. It was observed that rutin played a key role in the chronic phase [2]. Diosmin and hesperidin could reduce Leukotriene B4 (LTB_4_) synthesis and thereby reduce colitis in a trinitrobenzenesulfonic acid (TNBS)-induced colitis rat model [37]. Naringenin also shows anti-inflammatory activity by reducing leukocyte infiltration and the inhibition of pro-inflammatory cytokines such as IL-6, IL-8, TNF-α, IL-1β, IL-12, IL-4, IL-5, IL-13, IL-17, and IL-22 (Figure 3). It can also selectively block Nav 1.8, a sodium ion channel, thereby blocking sodium influx. Chalcones such as trans-chalcone, hesperidin, and methyl chalcone also showed inhibitory activity on pro-inflammatory cytokines [4]. The study conducted by Nam et al. (2017) showed common buckwheat sprouts containing flavonoid vitexin, orientin, and rutin. Tartary buckwheat sprout (TBS) containing a high concentration of rutin reduced NO production by inhibiting iNOS and COX2 expression in Lipopolysaccharide (LPS)-induced RAW 264.7 cells (Table 3) and also in male BALB/c mice. TBS was more potent not only in reducing NO but also in cytokines including IL-6, TNF-α, and IL-12. TBS extracts also reduced NF-κB and MAPK pathways by modulating on ERK, p38, MKK4, and JNK phosphorylation (Figure 4) [38]. Quercetin and myricetin could protect 661W cells and the cone photoreceptor cell line from the toxic effects of H_2_O_2_. They also enhance gene expression of M and S opsins under oxidative stress [39].

*Tabernaemontana catharinensis* A. DC. is an anti-inflammatory, analgesic, anti-ophidic folk medicinal plant. It was reported that intraperitoneally injection of the plant extract could reduce carrageenan-induced paw oedema in rats [40,41]. In a study conducted by Marques et al. (2018), the pretreatment with 100 mg/kg leaf extract of *T. catharinensis* inhibited oedema formation by 97.8% in rats. In the same study, the post-treatment of 50 mg/kg of EtOH extract also showed 62% reduction in oedema. The leaf extracts of this plant showed many flavonoids and phenolic compounds such as kaempferol, quercetin, and isorhamnetin [42]. A myeloperoxidase (MPO) assay conducted in a study showed a reduced levels of MPO when treated with the hydroethanolic extract of *T. catharinensis*, indicating a reduction in migration of polymorphonuclear (PMN) cells [42]. Hydroethanolic extracts of *T. catharinensis* leaves at 100 mg/kg body weight could reduce TNF-α and IL-1β. The EtOH extract of white mulberry fruits showed good antioxidant properties. It reduced ROS levels in LPS-stimulated RAW 264.7 macrophages. It was able to enhance anti-oxidative enzymes such as superoxide dismutase (SOD), chloramphenicol acetyltransferase (CAT), and plasma glutathione peroxidase (GSH-Px) and further reduced the expression of inducible NO synthase (iNOS) and nitrites in the cell, thereby protecting cells. It was further identified that the extracts contained flavonoids such as quercetin, kaempferol, luteolin, astragalin, and taxifolin [43].

## 6. Mechanism of Action of Flavonoids

Although the exact mechanism of flavonoids has yet to be studied, it is known that it exhibits anti-inflammatory properties by targeting many pathways. Different flavonoids have their unique way of reducing inflammation; many studies have concentrated on different types of flavonoids and their mode of action.

Quercetin showed its anti-inflammatory activity by inhibiting the c-Jun N terminal kinase and extracellular signal-regulated kinase, thereby inhibiting MAPK and AP-1 and NF-κB activity. Catechins also inhibited MAPK, AP-1, and NF-κB by inhibiting c-Jun N terminal kinase and p38 kinase (Figure 4). This implies that flavonoid targets the MAPK pathway and AP-1 transcription factor for decreasing inflammatory reactions [1]. It is also reported to decrease neutrophil recruitment and modulate actin polymerization in neutrophils [4]. Quercetin also could activate cyclic guanosine monophosphate (cGMP)/Protein kinase G (PKG)/adenosine triphosphate (ATP)-sensitive potassium channels pathway, leading to the hyperpolarization of nociceptive neurons, similarly to morphine and dipyrone, which is required in pain reduction [44,45]. This also reduced protein kinase C epsilon type (PKCε) and transient receptor potential cation channel subfamily V member 1 (TRPV1) in the spinal cord and DRG of paclitaxel-induced peripheral neuropathy rats and mice [46]. In quercetin and myricetin, the 3′-OH of the B ring interacts with PI3K and thereby attenuates the PI3K/Akt pathway (Figure 2) [4].

Apigenin reduced the levels of microRNA (miR33) and Toll-like receptor (TLR-4), NF-κB p65 pathway, leading to an increase in the ATP binding cassette A1 (ABCA1), which reduced lipid accumulation and also lessened muscle cells and macrophages in the atherosclerosis region of LPS-challenged apo-/- mice [11]. In TNF-α-treated human umbilical vein endothelial cells (HUVECS), isoliquiritigenin reduced the expression of NF-k-B inhibitor alpha (IkB-α), E-selectin, THP-1 monocyte adhesion, VCAM-1, and platelet endothelial cell adhesion molecule-1 (PECAM-1) by inhibiting NF-κB. It was also observed in the angiotensin II-induced hypertensive model; isoliquiritigenin reduced TNF-α and IL-1β; hence, extracellular deposition was reduced. In addition, the apoptosis induced by oxidative stress via nuclear factor E2-related factor (Nrf2) and NF-κB pathway was attenuated (Figure 2) [11].

Rutin decreased the levels of IL-6 and TNF-α by down-regulating NF-κB and ERK1/2 pathways (Figure 2); this also decreased VCAM-1 and ICAM-1 expressions in the high mobility group box 1 (HMGB) in induced HUVECS [11]. This also activated cGMP/PKG/ATP-sensitive potassium channels pathway and Nrf2/hemeoxygenase (HO) pathway and inhibited the NF-κB pathway to reduce pain and pro-inflammatory cytokines TNF-α and IL-1β (Figure 2) [4,47].

Silymarin reduced hypoxia-inducible factor-1α (HIF-1α) and inducible nitric oxide synthase (iNOS) by inhibiting NF-κB. The major component of silymarin, silibinin, is known to decrease epidermal growth factor receptor (EGFR), thereby reducing hypertrophy. In a study conducted by Iio et al., they showed that quercetin and baicalein inhibited the glyoxalase-I enzyme, which is known to have inflammatory properties by releasing histamine [48]. Luteolin, quercetin, kaempferol, and apigenin are shown to reduce the secretion of glucuronidase-1 and lysozyme enzymes from neutrophils [2].

Genistein is shown to affect the tyrosine kinase pathway and exhibit anti-inflammatory properties. They inhibited the activity of p56 lck, a T-cell protein kinase involved in IL-2 and IL-2R expression, hence reducing the cytokines in T-cells stimulated by phytohaemagglutinin (PHA)/phorbolmyristate acetate (PMA) [2]. Luteolin reduces inflammatory responses in alveolar macrophages by attenuating NF-κB and AP-1 pathways. Genistein, kaempferol, quercetin, and daidzein not only inhibited NF-κB also inhibited the Signal transducer and activator of transcription 1 (STAT-1) activation, thereby having higher potency to decrease NO synthesis [2].

Apigenin was observed to alleviate interferon-gamma (IF-γ) in vivo. It was also shown that flavonoids reprogram a pro-inflammatory response to anti-inflammatory in LPS- or IF-γ induced cells [49]. Flavonoids also enhance natural killer (NK) cells and cytotoxic T cells activity. A study conducted by Ruiz-Iglesias et al. (2020) shows that hesperidin modulated systemic immunity in rats that underwent high training and exercise [50]. Fisetin inhibits the phosphorylation of MAPK and translocation of NF-κB to reduce cytokine secretion in phorbol-12-myristate-13-acetate plus calcium ionophore (PMACI) induced rats. Luteolin-8-C-fucopyranoside (LU8C-FP) also targets NF-κB and MAPKs pathways to reduce IL-6 [10]. In a study by Kang et al. (2010), luteolin from *Lonicera japonica* Thunb. inhibited ERK1/2, c-Jun N-terminal kinases (JNK)1/2, and NF-κB pathway and also inhibited IL-6, TNF-α, and COX2 expression (Figure 4) [51]. Andrographolide, a flavonoid isolated from *Andrographis paniculata* (Burm. f.) Nees, inhibited NF-κB via the STAT3 pathway in LPS-induced RAW264.7 cells [52]. Kaempferol present in *Folium eriobotryae* decreased iNOS expression and NF-κB activation [53]. Daidzein, genistein, isorhamnetin, kaempferol, quercetin, naringenin, and pelargonidin reduced iNOS expression and attenuated NF-κB activity. Daidzein, genistein, kaempferol, and quercetin also inhibited the STAT-1 pathway [54].

Vitexin could attenuate the receptor activator of nuclear factor kappa-Β ligand (RANKL)-induced activation of MAPK and NF-κB pathway and also the activation of the Nrf2/HO-1 pathway [55]. It further inactivated transient receptor potential cation channel subfamily V member 1 (TRPV1) expression and thereby could reduce pain in capsaicin-induced pain in in vivo studies [56]. Oroxylin A attenuates calcium STAT pathway, thereby reducing NO and cytokines in LPS induced mouse model [57].

In addition, flavonoids are also potent antioxidant molecules that impart anti-inflammatory activity. They induce transcription factors such as Nrf2, a antioxidant responsive element (ARE), which mediates the expression of antioxidant proteins. Nrf2 suppressed the expression of MCP-1 and VCAM-1 and thereby decreased monocyte adhesion and transmigration to endothelial cells, which reduced MAPK and p38 expression and inhibited the formation of atherosclerotic lesions in mice and rabbits [58]. Oroxylin A activates the Nrf2/ARE pathway and inhibits NF-κB [57]. Flavonoids also induce enzymes such as hemeoxygenases, superoxide dismutase, γ-glutamylcysteine synthetase, glutathione peroxidase, and glutathione reductase, which are antioxidants [1]. Many diseases such as Alzheimer’s disease, rheumatoid arthritis, type II diabetes, and some types of cancer are induced by hyper-inflammatory responses. Cytokines such as IL-1β, IL-6, and TNF-α are observed to be in high levels in these conditions [10]. Hence the anti-inflammatory activity of flavonoids helps in preventing many health disorders.

## 7. Effects of Flavonoids in Cardiovascular Disease

Cardiovascular diseases (CVDs) include hypertension, myocardial infarction, and atherosclerosis and are the major causes of death in most the developed countries [2,10]. Chronic inflammation plays a vital role in the beginning and progression of CVDs [11]. The activity of flavonoids against inflammation thereby shows beneficial effects in reducing CVDs. Quercetin, a flavonoid, inhibits the NF-κB and Akt pathways induced by LPS, resulting in a reduced level of IL-6, TNF-α, and IL-1β in neonatal rat cardiac fibroblast. Luteolin and rutin enhanced NO in vivo sodium fluoride-induced hypertensive model, thereby reducing kidney injury marker-1, NF-κB, and cardiac troponin-1 (cTn) and also reduced blood pressure. In neonatal rat cardiac myocytes, luteolin reduced the degradation of IκB-β, NF-κB translocation, and DNA binding; hence, TNF-α levels were maintained low [11].

Fisetin downregulates NF-κB and receptors for advanced glycation end products and, hence, reduces myocardial injury markers, TNF-α, IL-6, lactate dehydrogenase (LDH), creatine kinase-muscle/brain (CK-MB) in blood, and normalised the ultrastructure and histology of the heart. It also balanced anti- and pro-apoptotic genes and anti- and pro-oxidants in the myocardium. As NF-κB expression decreased, IL-6, TNF-α, and IL-1β levels are low; hence, the occurrence of diabetic cardiomyopathy decreases and maintains normal heart morphology and low-level cardiac function markers such as CK-MB, LDH, cTn [11]. In carfilzomib-induced cardiotoxicity rat, rutin up-regulated IF-κB-α and, hence, down-regulated NF-κB, which attenuated the heavy chain of β-myosin, reducing B type natriuretic peptide mRNA expression and, hence, providing protection from myocardial hypertrophy [59].

Chrysin, one of the flavones, is known to suppress vascular endothelial growth factors (VEGF), Akt, and NF-κB and MAPK pathway, which prevented rats from doxorubicin-induced cardiomyopathy [11]. In another study, chrysin reduced the right ventricular systolic pressure and mean pulmonary artery pressure and also decreased the expression of collagen I, collagen III, and NF-κB in a monocrotaline-induced pulmonary arterial hypertension [60]. Chrysin also reduced hemodynamic and ventricular dysfunction and myocardial ultrastructure damage in isoprenaline-induced myocardial injury in a rat model. It has been observed that chrysin increases peroxisome proliferator-activated receptor gamma (PPAR-γ), whereas it inhibits TNF-α and NF-κB. Studies have shown that chrysin could decrease fibrosis in interstitial and perivascular regions and collagen expression in myocardial infarction in rat models [11,61]. Genistein reversed the atherosclerotic damage caused by angiotensin-induced NF-κB, CRP, MMP-9, the phosphorylation of p38, and ERK1/2, and it also increased PPAR-γ [11].

## 8. Effect of Flavonoids in Type 2 Diabetes

Diabetes mellitus is one of the extremely common metabolic disorders in the growing world [62]. The disease is characterised by increased blood glucose levels leading to health complications, including nephropathy, CVDs, retinopathy, etc. [62]. The cause of the disease is either lower insulin production due to genetic mutation or hereditary, modification in habitual eating patterns, or insulin resistance caused by over secretion of insulin or chronic inflammations leading to autoimmune disorders [62]. Flavonoids not only act on the inflammatory pathway but also maintain blood glucose levels and reduce the risk of diabetes-related disease [62]. Quercetin showed an anti-inflammatory response in hypertriglyceridemia-related acute pancreatitis in rats by reducing TNF-α, IL-1β, NF-κB, and IL-6, hence reducing histopathological damage [10]. It is also shown to enhance the 5’ adenosine monophosphate-activated protein kinase (AMPK) pathway, thereby stimulating GLUT4 expression [62]. Animal studies have shown that a reduction in blood glucose levels was observed when animals were treated with 10, 25, and 50 mg/kg of body weight quercetin [63]. In a study, Chinese-consuming quercetin had lower chances of obtaining type 2 diabetes. It was also observed to reduce lipid peroxidation, glucose absorption by GLUT2, and a reduction in insulin-dependent PI3K activation was also observed. The regulatory effect on NF-κB also stimulated glucose-induced insulin secretion. They also inhibited tyrosine kinase inhibitors, which prevented diabetes [62].

Quercetin reduced glucokinase GLUT4 activity and also decreased hepatic gluconeogenesis and glycogenolysis and enhanced cell survival-related genes and the proliferation of liver in streptozotocin (STZ)-induced diabetic rats. Quercetin with sitagliptin improved β-cell function, glycemic control, metabolic profile, oxidative, and inflammatory activities in STZ-induced diabetic rats [62]. Rutin is one of the most effective flavonoids against diabetes. It is shown to reduce fasting blood glucose, improve glucose tolerance, and also reduce serum lipids more effectively. It is also observed to activate hepatic enzymes hexokinase, reduce gluconeogenesis, and improve lipid metabolism. Rutin treatment reduces glycosylated haemoglobin and fasting blood glucose levels in STZ-induced diabetic rats. Rutin was also observed to reduce caspase-3 activity by enhancing BCL-2 activity in the diabetic retina [62,64]. Kaempferol enhances adenosine monophosphate (AMP)-induced protein expression; suppresses apoptosis, thereby improving cellular viability and function. In a study, kaempferol decreased the RhoA/Rho kinase-induced pro-inflammatory pathway in normal rat kidney-52E cells (NRK-52E) and renal proximal tubule epithelial cells (RPTEC) [62]. Eriodictyol decreased the glucose-stimulated oxidative stress, inflammation, and cell viability by regulating the Nrf-2/HO-1 pathway. Hesperidin improved the Klotho/fibroblast growth factor-23 (FGF-23) pathway, thereby reducing insulin toxicity in liver cells. Apigenin regulated MAPK-NF-κB-TNF-α and transforming growth factor-beta-1 (TGF-β1)-MAPK-fibronectin signalling in STZ-induced diabetic nephropathy. Chrysin’s anti-inflammatory activity prevented STZ-induced diabetic mice from diabetic neuropathy. Baicalein activates the AMPK pathway and reduces insulin resistance and also modulates the PI3K/Akt pathway to reduce oxidative stress and inflammation in diabetic cardiomyopathy. They also regulate HMGB1/TLR4/NF-κB signalling, decreasing hepatic inflammation in diabetic mice [62]. Hence, the anti-inflammatory effect of flavonoids shows a protective effect against diabetic-induced disorders.

## 9. Effects of Flavonoids in Rheumatoid Arthritis (RA)

Rheumatoid arthritis, an autoimmune disease caused by the infiltration of immune cells such as T-cells, macrophages, fibroblasts, and B cells in the synovial membrane, leading to the destruction of joints and, hence, a loss of its function. The anti-inflammatory activity of flavonoids is known to have a role in reducing pain and inflammation in joints. Oroxylin A at 10 mg/kg body weight in type II collagen-induced arthritis (CIA) mice model reduced Th17 and enhanced T_reg_ cells and also inhibited TNF-α, IL-6, IL-17, and IL-1β; it also suppressed ERK1/2, MAPK, and NF-κB pathways [65,66]. Baicalin enhances Foxp3+ and T_reg,_ inhibits Th17 differentiation in vitro and in vivo [67]. In another study, baicalin reduced splenic Th17 cells in murine adjuvant-induced arthritis. Baicalin also reduced CIA in rat synovium via the reduction of IL-1β and TNF-α [68]. Baicalin containing UP446 could reduce RA by reducing TNF-α, IL-6, IL-17, and IL-1β. The combinations of flavonoids derived from *Scutellaria* roots are able to reduce PGE2 [69]. Icariin, a prenylated flavonoid, blocks the STAT3 pathway, hence reducing IL-17 and Th17, which reduces the cartilage and bone degradation in CIA mice [70]. Apple is rich in flavonoids such as procyanidin and condensed tannins. It was shown that the supplementation of condensed tannins from apples showed delayed arthritis symptoms in DBA1/J mice with CIA [71]. It was also observed in a study that procyanidin in apples could reduce IFN-γ and IL-17 [69]. Grape seed extract containing proanthocyanidin reduced TNF-α, thereby reducing CIA. It was also observed to reduce osteoclastogenesis in vitro [72].

## 10. Effects of Flavonoids in Neurodegenerative Diseases

Flavonoids are also known to have neuroprotective properties. Flavonoids interact with glial signalling and other cellular pathways, hence enhancing neuronal function and also impacting neuronal regeneration [73]. Flavonoids in blueberry activate the ERK pathway, which enhances cAMP response element-binding protein (CREB); hence, the brain-derived neurotrophic factor (BDNF) is up-regulated in the hippocampus of a rat. CREB activation by flavonoids is impactful in the regulation of memory and synaptic plasticity. Catechins are used in the green tea-modulated protein kinase A/CREB pathway to reduce amyloid beta 42 (Aβ_42_) oligomers. Flavonoids also stabilise Nrf-2, hypoxia-inducible factor-1, to regulate PPAR-γ and the activation of PPAR-γ coactivator 1-alpha (PGC-1α), to reduce oxidative stress, and improve mitochondrial dysfunction; thereby, Alzheimer’s progression is attenuated. Quercetin binds to the ATP-binding pocket of PI3K and inhibits its activity, thereby activating pro-survival pathways. Hesperetin is observed to activate the Akt/protein kinase B pathway to up-regulate pro-survival signals in cortical neurons. ECG elevates the phosphorylation of CREB via ERK and PI3K pathway, which enhances glutamate receptor-2; hence, neurotransmission, synaptogenesis, and plasticity are modulated. Wogonin reduces Aβ accumulation in SH-SY5Y cells via the mammalian target of rapamycin (mTOR) pathway [73].

Apigenin decreased mRNA expression levels of TNF-α, IL-1β, and IL-6 in subarachnoid haemorrhage rats [10]. Baicalin reduced Th17 and Th1 cell differentiation via STAT/NF-κB signalling, thereby alleviating the severity of autoimmune encephalomyelitis (EAE) [74].

## 11. Effects of Flavonoids in Retinal Degeneration

Retinal degeneration due to mutations, excessive light exposure, and also inheritance is the major cause of blindness. Rhodopsin and cone opsins are major photoreceptors that are required for sight. Excessive light results in the degeneration of photoreceptors by enhancing ROS and pro-inflammatory cytokines and induces apoptosis. Therapeutics aims to reduce inflammation, oxidative stress management, and anti-apoptotic pathways acceleration. Many flavonoids are shown to have all these properties. It was observed that the intraperitoneal injection of quercetin or myricetin at 20 mg/kg for ATP-binding cassette subfamily member 4 (Abca4^−/−^) retinol dehydrogenase 8 (Rdh8^−/−^) mice 30 min before exposure to 10,000 Lux light for 45 min showed a protective effect against light-induced retinal degeneration by maintaining retinal morphology and function [39]. The treatment of quercetin or myricetin at 20 mg/kg to BALB/c mice also showed a positive effect on retinal protection. It was observed that dimethyl sulfoxide (DMSO)-treated mice activated microglia formation and inflammatory reactions after exposure to light; in contrast, in quercetin or myricetin-treated mice, the attenuation of these pathways was observed. The functions of DMSO-treated and light-exposed mice were recovered by flavonoid treatment. Myricetin could reduce ROS in light-induced Abca4^−/−^Rdh8^−/−^ mice but could not eliminate it completely. However, inflammatory cytokines such as the CC motif chemokine ligand-2 (CCL2), IL-6, and TNF-α were completely decreased by the treatment of either quercetin or myricetin in light-induced Abca4^−/−^Rdh8^−/−^mice. It was also observed that flavonoids could halt the expression of BAX via balancing BAX/BCL-2 in Abca4^−/−^Rdh8^−/−^ mice [39].

Fisetin is also known to enhance myocyte enhancer factor 2c (Mef2c) [75], which induces the gene expression of photoreceptors. It was also shown in a study that the flavonoid-treated cells increased the gene expression of rhodopsin, M, and S opsin by 2–3, 4–5, and 3.5–4.5-fold three days after illumination and further increased on the seventh day [39].

## 12. Effects of Flavonoids in Inflammatory Bowel Disease (IBD)

Inflammatory bowel disease (IBD) represents a group of intestinal disorders that cause the prolonged inflammation of the digestive tract. This disease is characterised by abdominal bleeding, pain, diarrhoea, and loss of appetite [76]. Flavonoids show a beneficial effect in reducing inflammation. Rutin was able to reduce cytokines such as IL-1β and reduce inflammation in the colon in trinitrobenzene sulfonic acid (TNBS) induced colitis. It has been found that 0.1% of rutin in the diet for two weeks reduced the dextran–sulphate sodium (DSS)-induced colitis [76]. Quercetin reduced iNOS expression via the NF-κB pathway in DSS-induced colitis. Quercetin of 50–100 mg/kg maintained the GSH levels in acetic acid-induced colitis. Morin also reduced intestinal inflammation by acting on colonic leukotriene B4 (LTB4), IL-1β, and NO. Kaempferol inhibits the NF-κB pathway to reduce IL-6, IL-1β, COX2, NOS, TNF-α, and reduces DSS-induced colitis [77]. Isoflavones act as oestrogen receptors and use signalling to reduce TNBS-induced colitis. Fermented soy gram, consisting of isoflavones, down-regulated IL-1β and up-regulated IL-10; hence, the permeability of intestinal cells decreased in TNBS-induced colitis. Isoflavones also reduced IL-8 via TNF-α inhibition in Caco-2 cell lines. Genistein is also known to mimic oestrogen and reduce IBD symptoms. Moreover, genistein inhibited MPO and COX2 activity via the NF-κB pathway. Daidzein is one of the isoflavones that reduces IL6-, IL-8, IL-12, INF-γ, and up-regulates IL-10 in mesenteric lymph node cells in DSS-induced colitis [77].

Naringin decreased xanthine oxidase, alkaline phosphatase, MPO, malondialdehyde (MDA), and NO. They also downregulated iNOS, ICAM-1, MCP-1, IL-6, MIP-2, PGE_2_, INF-γ, and IL-17A. This reduced DSS-induced colitis. Hesperidin also reduced colitis by attenuating MDA, MPO, and IL-6. Anthocyanins also show anti-inflammatory properties and reduce pro-inflammatory cytokines. Anthocyanins reduce IBD by minimising the expression of IL-6, IL-9, INF-γ, MPO, TNF-α, IL-1β, IL-17A, iNOS, and COX2. Apigenin and luteolin decreased IBD by regulating TNF-α, IL-1β, iNOS, and COX2 expression. Luteolin was also observed to reduce CD4^+^ T cell infiltration. Diosmin acted on LTB4 MPO to increase GSH levels. Wogonin and tangeretin increased claudin-1 zonula occluden-1 in the tight junctions. They also reduced IL-6, IL-1β, IL-8, iNOS, and COX2; and TLR4, MyD88, TGF-β-activated kinase 1 (TAK1), IL-23, and TNF-α expression. Tangeritin also decreased T helper cells in Th1 and Th17 differentiation [77].

## 13. Effect of Flavonoids in Cancer Treatment

Inflammation is responsible for controlling many cellular pathways, for which its unregulated expression leads to cancer. Hence, inflammation is a hallmark of cancer initiation and progression. The anti-inflammatory property of flavonoids also reduces cancer progression; hence, flavonoids show anti-cancer activities as well. Hesperidin was seen to decrease cell viability in the C6 glioma cell line [5]. Flavonoids are known to inhibit TNF-α, which otherwise would induce MCP-1/CCL2 release; this further enhances the infiltration of tumour-associated macrophages (TAMs), myeloid-derived suppressor cells (MDSCs), T_regs_, metastasis-associated macrophages (MAMs), tumour-associated neutrophils (TANs), and Th17 cells, which are required for maintaining tumour microenvironments. Flavonoids also inhibit fibroblast differentiation into cancer-associated fibroblast by targeting TGF-β2 [78]. In a study by Hou et al. (2019), flavonoids target TNF-α, and IL-1β to reduce inflammation and, hence, inhibit recurrent colitis and colorectal cancer [79]. The flavonoids are also known to attenuate many signalling pathways relating to inflammation and cell proliferation, which include MAPK, NF-κB, ERK1/2, mTOR, PI3K, and Akt pathways. Flavonoids are studied to decrease C-X-C motif chemokine receptor 4 (CXCR4) expression, which reduces metastasis. It also modulates the Integrin-linked protein kinase (ILK)/Yes1-associated transcriptional regulator (YAP) pathway to reduce Epithelial-mesenchymal transition (EMT) and metastasis [78]. *Ageratum conyzoides* L. showed a cytotoxic effect against mouse leukaemia cells and human non-small lung cancer cells [80]. It also inhibited the growth of glioblastoma cell lines and prostate cancer cell lines [81,82]. Flavonoids in *A. conyzoides* inhibit the proliferation of HeLa cells by inducing S phase arrest in the cell cycle. They also induced apoptosis in HeLa cells [81]. Many flavonoids reportedly arrested the cell cycle at G0/G1 phase or G2/M phase transitions [83]. Flavonoids from Chinese bayberry induced G1 phase arrest in ovarian cancer cells [84]. The flavonoids of *Citrus platymamma* Hort.et Tanaka showed the growth inhibition of A549 human lung cancer cells by arresting the cell cycle at G2/M phase [85]. Licochalcone 2′,4′-Dihydroxy-6′-methoxy-3′,5′dimethylchalcone is the flavonoid that enhanced intracellular ROS levels to activate apoptotic pathway in bladder and hepatocellular cancer cells [86,87]. Further reports suggest that luteolin and quercetin could attenuate EMT, thereby reducing the progression of cancer in squamous carcinoma cells [88]. Isoliquiritigenin, a flavonoid from licorice, inhibited EMT in ovarian cancer cells [89]. In a study, cells treated with flavonoid from *A. Conyzoides* showed enhanced E-cadherins and reduced N-cadherins and vimentin in xenograft tumours, thereby inhibiting invasion and migration of HeLa cells [81].

## 14. Effects of Flavonoids in Obstructive Pulmonary Disorders

Chronic inflammation in the lungs due to gases or noxious particles leads to a reduction in the function of the lungs, thereby leading to persistent breathing difficulty. The anti-inflammatory activity of flavonoids prevents inflammatory effects on the lungs. *Morus alba* (L.) extracts were able to reduce inflammatory cytokines such as IL-6, TNF-α, and NO in lung macrophages, thereby inhibiting epithelial hyperplasia and alveolar space destruction. Studies show that flavonoids enhance the expiratory force volume in one second and reduce chronic coughing, phlegm, and shortness of breath [17]. In a study, pulmonary emphysema was reduced in the chronic obstructive pulmonary disorder (COPD) model when treated with quercetin [17]. Tricetin and fisetin could reduce IL-6, TNF-α, IL-10, IL-8, and also poly [ADP-ribose] polymerase 1 (PARP-1), thereby reducing COPD [90]. Flavonoids such as kaempferol, myricetin, and quercetin could block elastase enzymes, which are produced by neutrophils and can enhance elastin and fibronectin, leading to many diseases such as pulmonary emphysema, respiratory distress syndrome, and acute respiratory distress syndrome (ARDS). Quercetin was efficient in reducing mucin 5AC (MU5AC), epithelial growth factor receptor (EGFR), pPKC4-hydroxynonenal (HNE)-induced protein expression, and also the phosphorylation of ERK1/2, thereby reducing mucous secretions in human airway epithelial cells [17]. Quercetin also inhibited serine/threonine kinase and tyrosine kinase and enhanced type III deacetylase Sirt-1 protein to down-regulate matrix metalloproteinases (MMP)-9 and MMP-12. It also increased the elasticity of the lungs. Liquiritin apioside is a flavonoid that reduces cytotoxic effects in cigarette smoke-induced oxidative stress in human epithelial cell lines by reducing TNF-α and TNF-β. Flavonoids reduced pulmonary neutrophils and macrophage inflammation in ICR mice, and it also reduced mucous goblet cells. Baicalin could reduce TNF-α, IL-8, and MMP-9 in COPD BALB/C mice [17]. Vitexin could induce the Nrf/HO-1 mediated antioxidant pathway in LPS-induced acute lung injury model [91]. Icariin reduces CD4^+^ROR*γ*t^+^ T cells and enhances CD4^+^Foxp3^+^ T cells in bronchial-alveolar lavage fluid (BALF) [92]. Flavonoids markedly reduced lung wet-to-dry ratio pulmonary edema in BALB/c mice. Flavonoids such as luteolin, morin, fistein, and scutellarein show positive effects on asthma by targeting many inflammatory cytokines including IL-4, IL-13 TNF-α histamine, and also enzyme phospholipase A2 and pathways such as 2-STAT1/3 and EPO (eosinophil peroxidase) activity [17].

## 15. Effect of Flavonoids in Coronavirus Disease 2019 (COVID-19)

The hyper-inflammatory response causes severity in SARS-CoV-2 infection. SARS-CoV-2 binds to angiotensin converting enzyme-2 (ACE-2) receptor, which activates angiotensin II, which further activates pro-inflammatory cascade via NF-κB and MAPK pathway. Several flavonoids, including nepitrin and hesperidin, attenuate angiotensin and contribute to reducing inflammation caused by COVID-19. In silico studies on caflanone a flavonoid-based phytomedicine, show that it inhibits TNF-α, IL-1β, IL-8, IL-6, and macrophage inflammatory protein-1-alpha (Mip-1α). Hesperidin is one potent flavonoid with anti-viral, anti-oxidant, and anti-inflammatory properties, which is also observed decrease IL-33 and TNF-α in LPS-treated mice model. This can be a good candidate for COVID-19 treatment, although further studies are required. Rhamnocitrin, a flavonoid present in *Nervilia fordii* (Hance) Schltr, is observed to inhibit vascular endothelial activation, which decreases cytokines. This can also be used to reduce the cytokine storm in the COVID-19 disease. Meanwhile, studies have shown traditional Chinese medicine, which mainly has flavonoids, down-regulated ACE-2 expression. Flavonoids also modulate inflammatory mediators such as TLR and NLR family pyrin domain containing 3 (NLRP3) inflammasomes, which are known to have a prominent role in SARS-CoV-2 pathology. Fisetin, amentoflavone, and isoliquiritigenin are putative ligands which bind to the bromodomain containing protein 4 (BRD4) receptor, which otherwise is bound by transmembrane protein E of SARS-CoV-2. This inhibits NF-κB mediated inflammatory response. Xanthohumol reduces LPS-induced lung injury by activating the AMPK/GSK3β-Nrf2 pathway. Flavonoids from *Crateva nurvala* Buch. Ham, *Apios americana* Medikus leaves, and EGCG activated the Nrf2 pathway and decreased inflammation in endothelial cells, RAW264.7 cells, and in in vivo models. The flavonoid 6-demethoxy-4′-O-methylcapillarisin derived from *Artemisia rupestris* L. activated the Nrf2/hemeoxygenase pathway [46,49]. Epicatechin, EGCG, and chrysin are natural flavonoids that inhibit dipeptidyl peptidase 4 (DPP4), which is potent to cause cytokine storms in SARS-CoV-2 infection. Quercetin, scutellarin, and myricetin are good 3-chymotrypsin-like protease (3CL^pro^) inhibitors, which are crucial viral proteins for viral maturation [49].

## 16. Flavonoids under Human Trials

Although there are many studies on flavonoids as an anti-inflammatory compound, there are not many studies using human candidates. However, there are a few studies where whole-plant extracts are used in human studies. The human trials on flavonoids as a treatment are briefed in the following paragraph. The administration of green tea extract, green tea, and black tea for four weeks showed a decrease in P-selectin levels and increased urinary 4-*O*-methyl gallic acid. Catechin present in cocoa tablets also showed the same effect on P-selectin levels when administered for four weeks. Six weeks of administration of black tea decreased CRP levels, although it was not observed in coronary artery disease patients in a different trial [1]. There was a decrease in the levels of IL-1α, CRP, fibrinogen, VCAM-1, and ICAM-1 in plasma and increased levels of epigallocatechin, when red wine was administered for 32 hemodialysis patients for four weeks [93]. Red wine also reduced NADH oxidase activity in neutrophils and reduced TNF-α in 20 postmenopausal women [94]. Soya supplementation showed reduced VCAM-1 levels in hemodialysis patients in a long-term intervention study. Quercetin supplementation to coronary artery disease patients showed a decrease in chronic systemic inflammation markers [1].

A study in the USA with 200,000 men and women has showed that consuming apples, pears, and blueberries containing anthocyanin reduced the risk of diabetes [62]. Hesperidin 500 mg daily for three weeks supplementation to 24 candidates reduced circulating inflammatory cytokines and increased flow-mediated dilation [95]. In another study, 500 mg of daflon for a week reduced oedema, congestion, and inflammation in 100 candidates [96]. It also reduced pain, bleeding, and mucosal discharge when 105 candidates have been treated with 500 mg daflon for four weeks [97]. A dosage of 379 mg of green tea extract for three months improved blood pressure, insulin tolerance, lipid profile, inflammation, and oxidative stress [98]. Silymarin at 420 mg daily dose for 90 days reduced joint swelling, pain, and tenderness in 44 candidates [99]. Three weeks of treatment of 220 mg Pycnogenol^®^ reduced C-reactive protein and inflammation in 67 osteoarthritis patients [100]. In another study, 100 osteoarthritis patients showed pain relief and reduced stiffness when 150 mg of Pycnogenol^®^ was administered daily for three months [101]. Flavopiridol also reduced tumour burden in 10 chronic lymphatic leukaemia when administered at 30 mg/m^2^/min daily and 4 h infusion of 30 mg/m^2^/week for 3 weeks in every 5 weeks; this cycle was repeated twice [102]. Green tea consumption reduced tumour progression in prostate cancer patients and also reduced the risk of lung cancer [103,104]. Eight weeks of supplementation of 500 mg quercetin reduced early morning stiffness pain in 50 RA patients [105]. A total of 13,651 adults were supplemented with catechins, flavonols, and flavones for four years and examined for COPD symptoms and pulmonary function; catechin could reduce all symptoms of COPD, and flavonols and flavones could reduce cough only [106].

## 17. Conclusions

Fruits and vegetables are rich in flavonoids and possess many health benefits. Most of the flavonoids target NF-κB, MAPK, ERK, and Akt pathways to reduce inflammation, oxidative stress, and thereby many diseases. Many flavonoids are able to reduce inflammatory cytokines such as TNF-α, IL-6, IL-8, IL-1β, IL-17, and IFN-γ. They are also efficient in reducing enzymes such as iNOS, COX2, glucuronidase, and lysozyme. They act on apoptosis and cell viability, leading to enhanced Nrf2 and AMPK pathways and antioxidant enzymes such as glutathione-S-transferase (GST), Heme-oxygenase-1, SOD, and CAT. These play a crucial role in alleviating inflammation-related diseases.

Hence, flavonoids can be one of the treatment options for many metabolic disorders, autoimmune diseases, neurodegenerative diseases, and also for many pulmonary diseases, including COVID-19 and post-COVID diseases. Several studies support the anti-inflammatory activity of flavonoids, and in vivo studies throw less light on activities. The major differences in the in vitro and in vivo studies are the flavonoid physiological concentrations. In vitro studies use a higher concentration of the compounds, whereas the in vivo bioavailability of a compound has less impact on inflammation. Extensive studies are essential for improving the bioavailability of flavonoids. Moreover, more human trials are required to demonstrate their capability as anti-inflammatory agents.

## Figures and Tables

**Table 1:** Different classes of flavonoids, their subtypes, structure, and sources of their isolation.

Flavonoids	Subtypes	Mol. Wt g/mol	Structure	Source	Reference
Flavanones	Naringenin	272.25	C_15_H_12_O_5_ 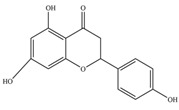	Citrus fruits	[1]
	Taxifolin	304.25	C_15_H_12_O_7_ 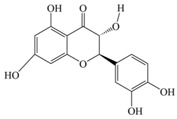		
	Eriodictyol	288.25	C_15_H_12_O_6_ 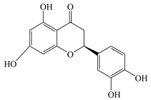		
	Hesperetin	302.28	C_16_H_14_O_6_ 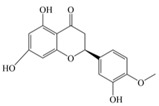		
Flavones	Apigenin	270.24	C_15_H_10_O_5_ 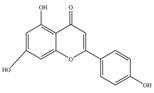	Sweet red pepper, parsley, chamomile, celery, mint, and *Ginkgo biloba*	[11]
	Wogonin	284.26	C_16_H_12_O_5_ 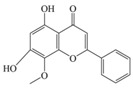		
	Luteolin	286.24	C_15_H_10_O_6_ 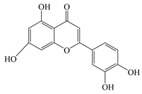		
Isoflavones	Genistein	270.24	C_15_H_10_O_5_ 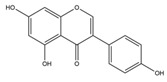	Tofu, roasted soya nuts, miso	[11]
	Daidzein	254.24	C_15_H_10_O_4_ 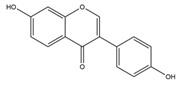		
	Glycetein	284.26	C_16_H_12_O_5_ 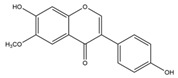		
Flavonols	Kaempferol	286.24	C_15_H_10_O_6_ 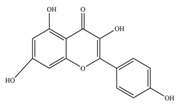	Saffron, lettuce, tomatoes, apples, grapes, berries, onions, kale, red wine, and tea	
	Myricetin	318.23	C_15_H_10_O_8_ 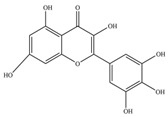		
	Fisetin	286.24	C_15_H_10_O_6_ 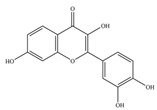		
	Silymarin	482.4	C_25_H_22_O_10_ 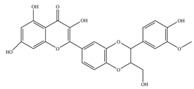		
	Rutin	610.5	C_27_H_30_O_16_ 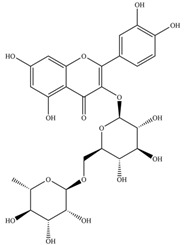		
	Isorhamnetin	316.26	C_16_H_12_O_7_ 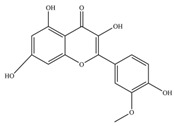		
	Quercetin	302.23	C_15_H_10_O_7_ 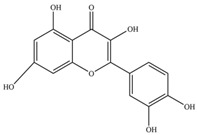		
Flavanols	Catechin	290.27	C_15_H_14_O_6_ 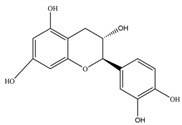	Black and green tea and fruits such as bananas, peaches, blueberries, apples, and pears	[11]
	Gallocatechin	306.27	C_15_H_14_O_7_ 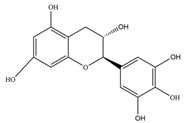		
	Epicatechin	290.27	C_15_H_14_O_6_ 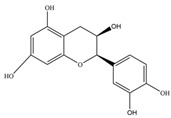		
	Epigallocatechin	306.27	C_15_H_14_O_7_ 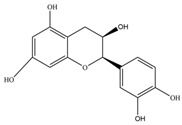		
	Epicatechingallate	442.4	C_22_H_18_O_10_ 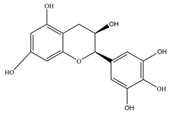		
	Epigallocatechingallate(EGCG)	458.4	C_22_H_18_O_11_ 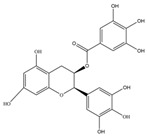		
	Procyanidin	594.5	C_30_H_26_O_13_ 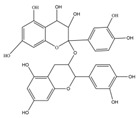		
Anthocyanin	Cyanidin	287.24	C_15_H_11_O_6_^+^ 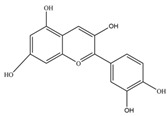	Merlot grapes, red grapes, raspberries, strawberries, blueberries, cranberries, bilberries, and blackberries	[11]
	Pelargonidin	271.24	C_15_H_11_O_5_^+^ 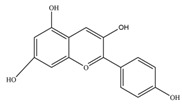		
	Malvidin	331.3	C_17_H_15_O_7_^+^ 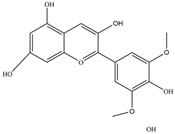		
	Delphinidin	338.69	C_15_H_11_ClO_7_ 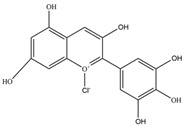		
	Peonidin	301.27	C_16_H_13_O_6_^+^ 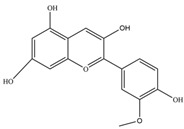		

**Table 2:** Some of the dietary sources of flavonoids.

Food/Dietary Source	Flavonoid	Quantity in mg L^−1^ (Approximately)	Reference
Green Tea	Gallocatechin B	383	[6]
	Epicatechin	738	
	Epigallocatechin	1565	
	Epicatechin-3-O-gallate	361	
	Kaempferol-O-glucoside	102	
	Quercetin 3-O-glucoside	185	
Black Tea	Quercetin 3-O-glucoside	119	[6]
	Kaempferol-O-glucoside	69	
Red Wine	Catechin	41	[6]
	Epicatechin	29	
	Anthocyanins	22	
Leek	Kaempferol	10-60 *	[6]
Onions	Anthocyanins	250 *	[6]
Potatoes	Anthocyanins	16300 *	[13]
Apples	Flavanols	91.7 *	[14]
Lemons	Flavanones	498.1 *	[14]

* These quantities are measured in mg/kg (it is mentioned in the Table header, mg/L).

**Table 3:** Mode of action of few flavonoids.

Flavonoids	Activity	Cells/Animal Model Used	Reference
Quercetin	Cyclooxygenase 2 inhibition	Rat peritoneal macrophages	[1]
	Inducible NO synthase inhibition	LPS/cytokine treated macrophages/cell lines	[1]
	Inhibiting MAPK, AP-1 DNA binding	LPS treated RAW cells.	[1]
	Extracellular signal-regulated kinase and p38 kinase inhibition	LPS treated RAW cells.	[1]
	Ob-Ra (leptin receptor), ERK1/2 phosphorylation, NF-κB, and TNF-α suppression	Leptin-induced human umbilical vein endothelial cells (HUVECS)	[11]
	Lysosomal enzyme reduction	Human polymorphonuclear leukocytes	[2]
	Neutrophils degranulation inhibition	Human neutrophils	[2]
Kaempferol	Cyclooxygenase 2	Rat peritoneal macrophages	[1]
	NF-κB inhibition	LPS treated macrophages	[1]
Apigenin	Inducible NO synthase inhibition	LPS/cytokine treated macrophages/cell lines	[1]
	NF-κB inhibition, TLR-4, Myeloid differentiation primary response 88 (MyD88), pI-κB-α reduction	LPS treated macrophages	[1,11]
	Cyclooxygenase 2	LPS treated macrophages	[2]
Luteolin	Inducible NO synthase inhibition	LPS/cytokine treated macrophages/cell lines	[1]
	NF-κB inhibition	Murine macrophages RAW 264.7	[2]
	TNF-α, IL-6 inhibition	IL-1_-induced human synovial sarcoma cells (SW982)	[10]
Luteolin-8-C-fucopyraNOSide (LU8C-FP)	IL-6 reduction	Phorbol-12-myristate-13-acetate plus calciumIonophore (PMACI)-stimulated THP-1 cells, human leukaemia monocytic cell lines.	[10]
Genistein	IL-6, TNF-α, IL-1β, IL-2, LTB4 inhibition	LPS treated Human blood monocyte	[1,2]
	NF-κB inhibition	LPS treated macrophages	[1]
	Cyclooxygenase 2	LPS treated macrophages	[2]
Epigallocatechin	NF-κB inhibition	LPS treated macrophages	[1]
Silybin	TNF-α inhibition	LPS treated RAW cells.	[1]
Rutin	nuclear factor E2-related factor (Nrf) activation and NF-κB inhibition	Human embryonic kidney reporter cell line	[11]
Wogonin	Cyclooxygenase 2	LPS treated macrophages	[2]
	TNF-α inhibition	LPS treated RAW cells.	[2]
Fisetin	TNF-α, IL-1β, IL-6, IL-8 reduction	Phorbol-12-myristate-13-acetate plus calciumIonophore (PMACI)-stimulated human mast cells	[10]

## Data Availability

The data presented in this study are available on request from the corresponding author.
